# Label-Free Homogeneous microRNA Detection in Cell Culture Medium Based on Graphene Oxide and Specific Fluorescence Quenching

**DOI:** 10.3390/nano11020368

**Published:** 2021-02-02

**Authors:** Florentin R. Nitu, Lorand Savu, Sorin Muraru, Ioan Stoian, Mariana Ionită

**Affiliations:** 1 Faculty of Medical Engineering, University Politehnica of Bucharest, Gh. Polizu St., No. 1-7, 011061 Bucharest, Romania; florentin.nitu@upb.ro (F.R.N.); sor.muraru@gmail.com (S.M.); 2 Molecular Biology Department, Genetic Lab, Milcov Street, No. 5, Sector 1, 012244 Bucharest, Romania; office@geneticlab.ro; 3 Royal Hospital, Splaiul Unirii Street, No. 313A, Sector 3, 030138 Bucharest, Romania; drstoianioan@royalhospital.ro

**Keywords:** graphene oxide, surfactants, fluorescence quenching, FRET, optical microRNA sensor

## Abstract

Label-free homogeneous optical detection of low concentration of oligonucleotides using graphene oxide in complex solutions containing proteins remains difficult. We used a colloidal graphene oxide (GO) as a fluorescent probe quencher to detect microRNA-21 spiked-in cell culture medium, overcoming previously reported problematic aspects of protein interference with graphene oxide. We used a “turn off” assay for specific quenching-based detection of oligo DNA-microRNA hybridization in solution. A fluorescein conjugated 30-mer single-stranded DNA (ssDNA) probe was combined with a complementary synthetic microRNA (18 nucleotides) target. The probe-target hybridization was detected by specific quenching due to photoinduced electron transfer (PET). On the next step, GO captures and quenches the unhybridized probe by fluorescence resonance energy transfer (FRET) in the presence of cell culture medium supplemented with platelet lysate, 0.1% sodium dodecyl sulfate (SDS), 0.1% Triton X-100 and 50% formamide. This resulted in sensitive measurement of the specific probe-target complexes remaining in solution. The detection is linear in the range of 1 nM and 8 nM in a single 100 μL total volume assay sample containing 25% cell culture medium supplemented with platelet lysate. We highlight a general approach that may be adopted for microRNA target detection within complex physiological media.

## 1. Introduction

Graphene is a two-dimensional nanomaterial with a single layer of carbon atoms arranged in a hexagonal lattice [1]. The applications of graphene and graphene derivatives cover a broad area including nano-electronic devices, energy storage, solar cells, metamaterials, optoelectronic devices [2,3,4], biomedical [5,6] and biosensor applications [7,8]. Several optical biosensors for oligo DNA [9,10] and microRNA detection are reported [11,12,13]. Such optical biosensors could serve as promising functioning principles for identification of diseases or other health anomalies.

Graphene oxide (GO) optical detection applications are based on fluorescently-labelled oligonucleotide probes as donors and graphene nanomaterials as quenchers [9]. The principle of fluorescence resonance energy transfer (FRET) is based on transfer of electronic excitation energy through dipole–dipole coupling of a fluorescent donor to an acceptor. The excitation energy absorbed by the donor molecule is transferred nonradiatively to an acceptor fluorophore or a quencher through dipole–dipole interactions through space (up to 10 nm distance). Transfer efficiency depends on distance between the molecules, donor and acceptor transition dipole orientation and spectral properties of the donor and acceptor. Contact or low range quenching (0–1 nm) by nucleobases from complementary strand is observed due to photoinduced electron transfer (PET), when single stranded oligo DNA labeled with a fluorophore is hybridized to an unmodified complementary strand [14,15,16]. For DNA biosensors it is generally considered that single stranded DNA (ssDNA) can stably adsorb onto GO due to π-π stacking interactions [17,18] and possibly, hydrogen bonding [19], whereas double stranded DNA (dsDNA) display lower affinities [9]. This makes GO a promising candidate for an optical detection device. However, GO has a tendency to gradually aggregate in complex media such as phosphate buffered saline (PBS), NaCl, and DMEM (Dulbecco’s modified Eagle’s medium) which contains ions, proteins and hydrophobic amino acids and promptly aggregates in cell medium and serum [20]. Our work aims to overcome these challenges by following a post-mixing protocol that would result in a well dispersed GO, leading to an optical biosensor based on specific fluorescence quenching. In turn, this would allow us to directly correlate the observed emission intensity to hybridization events, leading to a dependable accuracy of the results.

MicroRNAs are small, single-stranded, noncoding RNAs that are 17–25 nucleotides in length [21]. MicroRNAs have been reported to be present in exosomes [22] that are vesicles of endocytic origin released by many cells that have been identified in diverse body fluids. Serum miRNA can be free or exosomes-enclosed [23]. Circulating microRNAs (MiRNA) are reported as novel potential biomarkers for early diagnosis of acute or chronic diseases [24,25]. MiRNA are in fact present in clinical samples of plasma and serum in a remarkably stable form, displaying a concentration range from femtomolar to picomolar in normal physiologic conditions which can be significantly decreased or increased under pathological conditions [26].

Several studies investigated the oligoDNA and oligoRNA detection using fluorescence based sensors. Target DNA detection using GO sensor in diluted 1% serum assays was reported, with a linear response toward the target DNA concentration over the range from 0.4 nM to 5 nM [27]. The biosensor developed in this study achieved target detection of oligoDNA due to specific quenching in a homogeneous enzyme-free based on signal amplification. MicroRNA sensing in living cells based on peptide nucleic acid (PNA) and nano graphene oxide (PANGO) was reported [28,29]. Other method used DNA-silver nanoclusters in which the serum interference was minimized by using 100-fold diluted serum samples spiked with various concentrations of microRNA in the range of 2 pM to 200 pM. A linear dependence was obtained in the range from 1 pM to 20 pM with a detection limit of 0.7 pM [30]. Although GO was not part of this study, they specifically detect microRNA-21 through a label-free and homogeneous two-step protocol based on a hairpin-assisted cascade isothermal amplification method. A GO-based method has been developed for microRNA-141 detection by using rolling circle amplification (RCA). This assay is carried out by adding various concentrations of microRNA-141 into the 100-fold diluted healthy human serum samples [31]. In another report the serum was diluted with water 1/50 ratio and different concentrations of target microRNAs were prepared with serum media, with a sensitive detection of the microRNAs from 0.05 nM to 5 nM [32]. A homogeneous multiplexed microRNA FRET, amplification free assay was reported using 5% serum (7.5 μL serum samples in 150 μL total assay volume). The optical detection is based on time-gated Förster resonance energy transfer (TG-FRET), without use of GO [12].

**Figure 1:**
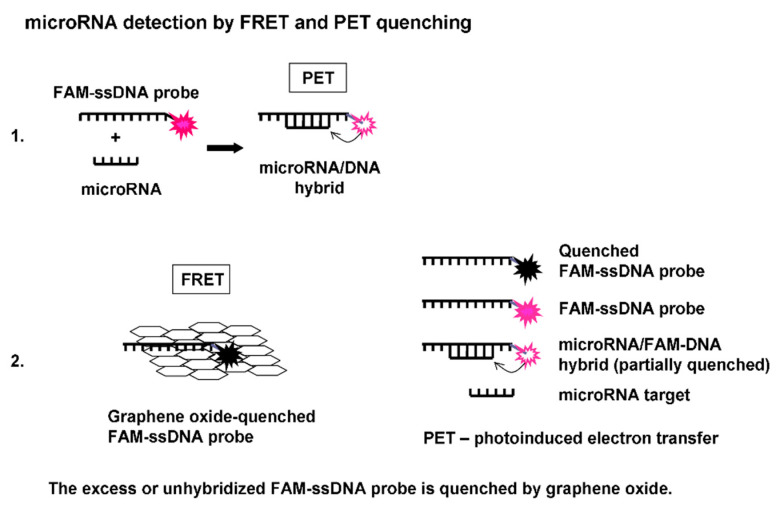
Schematic representation of the homogeneous detection assay.

In this work, we describe a homogeneous microRNA detection method in spiked cell culture medium supplemented with platelet lysate in the presence of sodium dodecyl sulfate (SDS) and Triton X-100 mixed micelles, based on two quenching fluorescence approaches. The first fluorescence quenching is obtained after hybridization reaction of DNA probe labeled by fluorescein and microRNA by photoinduced electron transfer (PET) obtained with the guanine present in the complementary microRNA strand. The second fluorescence quenching is obtained by adding the graphene oxide that preferentially attach single strand DNA rather than double strand and lead to quenching the fluorescence of fluorescein-DNA probe remains in solution (Figure 1). The surfactants stabilize GO dispersion in the presence of salts, improve hybridization by reducing oligonucleotide nonspecific binding, solubilize microRNA containing exosomes and SDS inactivates nucleases containing cell culture medium or serum. Also, we use formamide to decrease oligo DNA/microRNA hybridization temperature. The GO was noncovalent modified with the anionic surfactant SDS and bovine serum albumin (BSA) prior to be used in the assay. This assay uses specific hybridization quenching of fluorescein labeled single stranded DNA probe (FAM-ssDNA) in which the fluorophore is quenched by a guanine nucleotide from complementary strand. On the second step we used a GO post-mixing approach to quench unhybridized FAM-ssDNA probe [10,33,34]. Addition of GO results in adsorption and quenching by FRET of excess or unhybridized FAM-ssDNA probe and quantification of fluorescent FAM-DNA/microRNA hybrid proportional with the target concentration. A change in the fluorescence of these probes indicates the presence of a target nucleic acid, and there is no need to separate unbound probes from hybridized probes [35].

Fluorescein conjugated ssDNA probe hybridizes with microRNA (18 nt) in solution resulting FAM-DNA/microRNA hybrid with decreased fluorescence due to quenching by guanine from microRNA complementary strand. The unhybridized FAM-ssDNA probe is quenched by GO.

## 2. Materials and Methods

The following chemicals were purchased from Sigma-Aldrich, St. Louis, MO, USA: GO, 2 mg/mL, dispersion in H_2_O, cat. No. 763705. The graphene oxide carbon content (dry basis) is 42.0–52.0% and the oxygen content is 44–54%. This graphene oxide consists of monolayer sheets, with a mean sheet diameter: <10 μm, by laser diffraction (based on manufacturer specifications), magnesium chloride 98.0% (MgCl_2_), sodium chloride (NaCl), sodium dodecyl sulfate, Triton X-100, bovine serum albumin (BSA), formamide (molecular biology grade), ethylenediaminetetraacetic acid (EDTA). We used a nucleic acid oligonucleotide sequence based on a study of nucleic acid-GO interactions by Zhou et al. [36]. The DNA aptamer probe sequence was supplied by Integrated DNA Technologies, Inc. (Coralville, IA, USA), 3′-labelled with 6-fluorescein amidite (6-FAM), according to the sequence 5′-TTT CAA CAT CAG TCT GAT AAG CTA TCT CCC-3′/6-FAM (referred to as FAM-DNA). The corresponding single-stranded microRNA-21 (18nt) target molecule with complementary sequence 5′-UAG CUU AUC AGA CUG AUG -3′ (c-RNA) was purchased also from Integrated DNA Technologies. Cell culture medium consists of Minimum Essential Medium Eagle, #22561021 (ThermoFisher), supplemented with 5% (*v*/*v*) PLTMax Human Platelet Lysate, SCM141 (Sigma-Aldrich). The protein concentration in this cell culture medium is 2–3.25 mg/mL and the total concentration of hydrophobic aminoacids (L-Phenylalanine + L-Tryptophan + L-Tyrosine) is approx. 0.1 mg/mL.

Spectrofluorimeter measurements. The fluorescein-labeled single-stranded probe (5′-TTT CAA CAT CAG TCT GAT AAG CTA TCT CCC-3′/6-FAM) was hybridized with the complementary target oligo microRNA-21 (18nt) (c-RNA): 5′-UAG CUU AUC AGA CUG AUG -3′) in 10 mM Tris-HCl pH 8.0 buffer at 23 °C, before or after addition of 5 μg/mL or 40 μg/mL of GO. The components of the assay mix included: anionic surfactant SDS, with the linear formula: CH3(CH2)11OSO3Na, nonionic surfactant Triton X-100-polyethylene glycol tert-octyl phenyl ether, with the linear formula: t-Oct-C6H4-(OCH2CH2)xOH, x = 9–10, FAM-DNA probe and fluorescent microRNA/FAM-DNA hybrid, nonfluorescent microRNA target and quenched FAM-DNA probe. The commercial GO (2 mg/mL dispersion in water, Sigma-Aldrich) was homogeneously redispersed by sonication (Sonics Vibra Cell, VCX 750, 750W, 20 kHz) under ambient conditions for 10 min before use and then adjusted to 10 mM Tris-HCl pH 8.0 buffer during the assay. After incubation at room temperature, fluorescence emission intensity was recorded at 535 nm, with 5 reads per well option, using a Spark TECAN Fluorescence microplate reader. The adsorption kinetic was monitored by the microplate reader at 23 °C. Stock solutions were made fresh before the experiments. It was important to first add SDS surfactant to GO before BSA, in order to avoid aggregation and maintain GO dispersion. Oligo DNA and microRNA stock solutions were made containing 0.1% SDS. The GO stock solution contained 0.1% SDS and 0.1 mg/mL BSA. Precisely, 100 μL of final volume was used for all the samples distributed into Costar 96 well black, flat bottom plates, cat# 3915.

For complementary microRNA detection, several concentrations of FAM-ssDNA were used: 1 nM, 2 nM, 4 nM and 8 nM (L-1) with the same concentrations of microRNA (FAM-DNA: microRNA = 1:1), 1 mM EDTA 10 mM Tris-HCl buffer, pH 8.0; 10 μg/mL or 40 μg/mL of GO was added to the ssDNA or ssDNA + microRNA samples and the fluorescence signal was monitored. 

The hybridization assay in the presence of formamide was performed in assay mixture that contains 30%, 40% and 50% formamide, 0.1% SDS, 0.1 mg/mL BSA, 23 ºC and 42 ºC, 8 nM FAM-DNA. The microRNA (18nt): FAM-DNA molar ratio was 1:1, Figure 2. The same experiment was done in the presence of PicoGreen as an alternate method to detect hybridization (Supplementary Information, Figure S1). The experiments in Figure 3 and Figure 4 were performed in the presence of 25% cell culture medium supplemented with 5% platelet lysate, 50% formamide, 0.1% SDS, 0.1% Triton X-100, 5 μg/mL GO. The ionic strength of 25% cell culture medium is 40 mM. The experiments in Figure 5, were performed in the presence of 25% cell culture medium supplemented with 5% platelet lysate, 40% formamide, 1% SDS, 0.1% Triton X-100, 40 μg/mL GO, with addition of 50 mM and 100 mM NaCl. 

## 3. Results

### 3.1. MicroRNA (18 nt) Target Detection in the Presence of Formamide, BSA, SDS and NaCl

**Figure 2:**
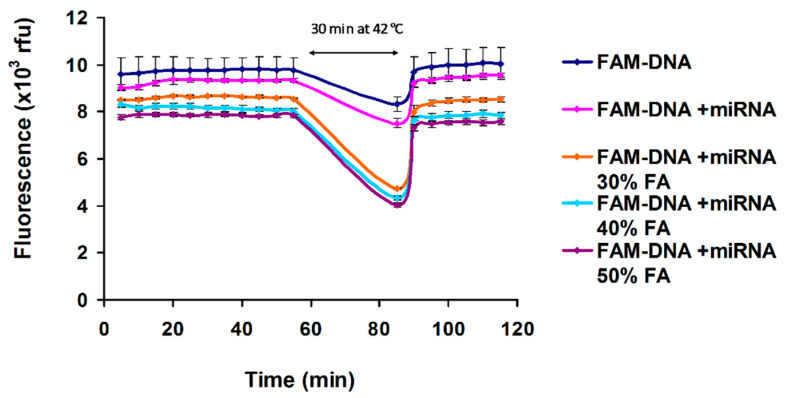
Effect of formamide on FAM-DNA/microRNA (18nt) hybridization. The detection is based on hybridization quenching due to photoinduced electron transfer (PET). The experiments were done in triplicate samples. Error bars represent the standard deviations of triplicate values.

**Figure 3:**
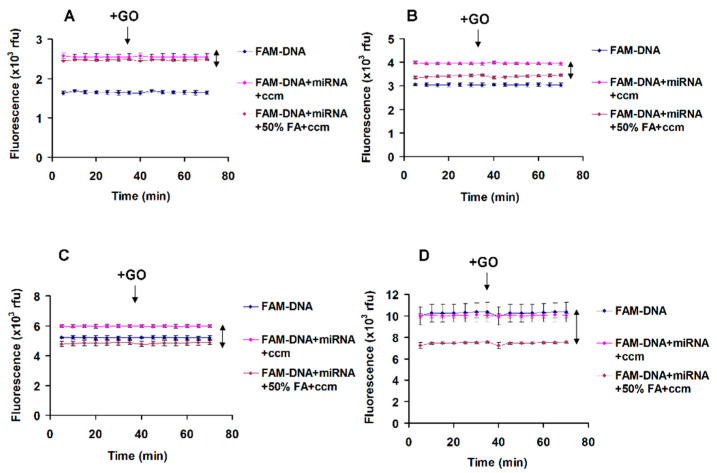
Detection of microRNA (18 nt) spiked-in cell culture medium (ccm). (**A**) 1 nM, (**B**) 2 nM, (**C**) 4 nM, (**D**) 8 nM microRNA. The microRNA detection is done by fluorescence quenching due to PET using complementary FAM-DNA fluorescent probe (double arrow). The experiments were done in triplicate samples. Error bars represent the standard deviations of triplicate values.

It was reported that GO sheets bind single stranded FAM-DNA probe [9] and single stranded microRNA target [37]. To increase detection sensitivity in a homogeneous assay, a “post-mixing” approach is used. The microRNA detection process is illustrated in Figure 1. On the first step, the microRNA target detection is based on specific fluorescence quenching of FAM-DNA probe by guanine from complementary strand [14,15]. After microRNA/FAM-DNA hybridization, excess/unhybridized FAM-DNA is quenched by GO. Using the two-step strategy of first performing solute probe-target hybridization (with quenching due to PET) before GO adsorption (with quenching due to FRET), we minimized the nonspecific binding of microRNA to GO. To demonstrate the functionality of the assay and check the specific PET quenching during hybridization between FAM-ssDNA (30 nt) and microRNA (18 nt), we design a homogeneous assay in complex conditions containing 0.1% SDS, 0.1 mg/mL BSA, 40 mM NaCl (similar with the ionic strength of 25% cell culture medium), 1 mM EDTA, 10 mM Tris-HCl buffer, pH 8.0 and various concentrations of formamide 30%, 40% and 50% to improve the hybridization yield, decrease the hybridization temperature [38] and protect RNA from degradation by RNases [39] (Figure 2). When FAM-DNA is mixed with GO in complex medium containing 40 mM NaCl, 1% SDS, 0.1% Triton X-100, 0.1 mg/mL BSA, its fluorescence is quenched (Figure 5A,B), but no quenching is observed in the presence of cell culture medium supplemented with platelet lysate (Figure 3).

The FAM-ssDNA and microRNA concentrations were 8 nM with the molar ratio 1:1. The data were acquired at 23 °C as a control background, during 30 min incubation at 42 °C and again at 23 °C. The hybridization is monitored using quenching by photoinduced electron transfer. The incubation at 42 °C was used to maximize the hybridization and the data were acquired continuously after the temperature reached again 23 °C (30 min incubation).

Figure 2, highlights the detection of microRNA (18nt) target in the presence of bovine serum albumin, SDS, NaCl and 30%, 40%, and 50% formamide concentration. We used formamide to decrease hybridization temperature and increase hybridization specificity [40]. A control of FAM-DNA + microRNA was used to establish quenching due to photoinduced electron transfer after hybridization in the presence of formamide. The amount of PET quenching due to hybridization increased with formamide concentration (Figure 2). The results shows that the hybridization is fast at 23 °C in the presence of formamide and the incubation at 42 °C does not change significant the quenching. Fast, 5 min, oligo DNA (22 nt) hybridization was reported using real-time Quartz Crystal Microbalance (QCM) in 1mM PBS containing 50% formamide, at room temperature [41]. In our experiment the highest hybridization was at 50% formamide as expected. This is a control experiment for the hybridization in the presence of cell culture medium. 

In order to check the FAM-DNA/microRNA hybridization in the presence of formamide by an alternate approach, we used the intercalating dye Pico Green to detect the DNA/microRNA hybrid. The data shows that the optimum binding of PicoGreen to FAM-DNA/microRNA hybrids is at 30% Formamide, 0.1% SDS and 0.1 mg/mL BSA. At lower formamide concentration hybridization is decreased and there is low PicoGreen signal, while at higher formamide concentration the binding of PicoGreen to double stranded hybrid microRNA-FAM-DNA is decreased (Figure S1, Supplementary Information).

### 3.2. microRNA-21 (18 nt) Target Detection in the Presence of Cell Culture Medium Supplemented with Platelet Lysate

The method described above was applied to the determination of microRNA-21 (18 nt) in spiked cell culture medium supplemented with platelet lysate samples.

Typically, microRNA-21 (18 nt) at various concentrations (1 nM, 2 nM, 4 nM and 8 nM) was added into the 25% diluted cell culture medium (100 μL total assay volume) supplemented with platelet lysate samples, and the fluorescence intensities were measured. The cell culture medium was pre-treated with 1% SDS and 0.1% Triton X-100 in order to solubilize colloidal exosome vesicles containing microRNA and to inactivate nucleases. The GO working stock solution was noncovalently modified with 0.1 % SDS, 0.1 mg/mL BSA and was prepared fresh before each experiment.

Figure 3, highlight the detection of microRNA (18 nt) target in the presence of 25% cell culture medium supplemented with 5% platelet lysate. There are four FAM-DNA concentrations: (A) 1 nM, (B) 2 nM, (C) 4 nM, (D) 8 nM. The GO, 5 μg/mL GO was added after hybridization (35 min incubation time required for hybridization) and then the signal was recorded. The FAM-ssDNA: microRNA (18nt) molar ratio was 1:1. The data were acquired at 23 ºC. The hybridization is monitored using quenching by PET. We notice that there is a fluorescence increase of about 1000 rfu (relative fluorescence units) due to cell culture medium autofluorescence (FAM-DNA + microRNA + ccm).

Upon second-step addition of GO to the FAM-DNA + microRNA + 50% formamide + ccm mixture, the original amount of fluorescence was quenched modestly, assuming that the hybridization was complete and just traces of single stranded FAM-DNA remains in solution.

**Figure 4:**
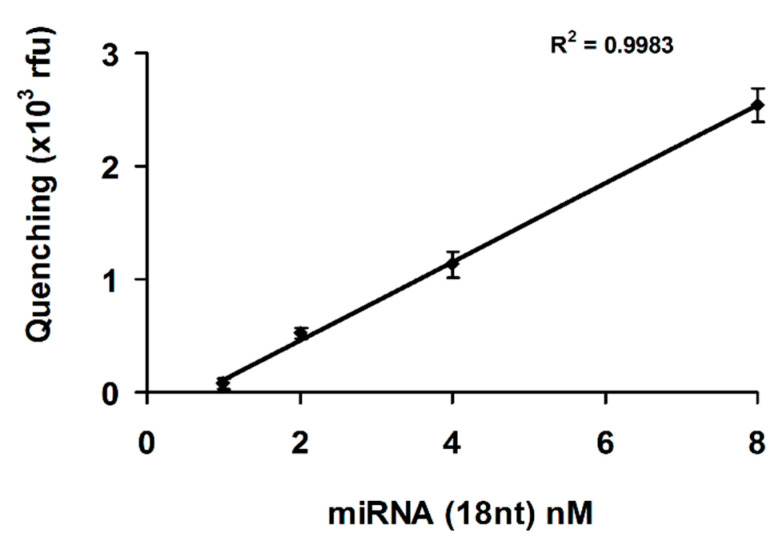
The FAM-DNA fluorescent probe was used in the presence of 25% cell culture medium supplemented with platelet lysate. GO was added after FAM-DNA/microRNA 18nt hybridization. The experiments were done in triplicate samples. Error bars represent the standard deviations of triplicate values.

Figure 4, highlight the quantitation of microRNA spiked-in cell culture medium, supplemented with platelet lysate. The plotted values are the difference between the control samples FAM-DNA + microRNA + ccm and the quenched samples due to hybridization in the presence of formamide FAM-DNA + microRNA + ccm + 50% formamide (Figure 3). The quantitation is linear in the range of 1 nM to 8 nM microRNA (18 nt) and the limit of quantitation (LOQ) is 1 nM microRNA (18 nt). 

### 3.3. Detection of microRNA (18 nt) Target in Cell Culture Medium in the Presence of Formamide and Salts

In order to increase quenching by GO, we used 50 mM and 100 mM NaCl concentration and increased surfactant concentration to maintain GO in dispersed form.

**Figure 5:**
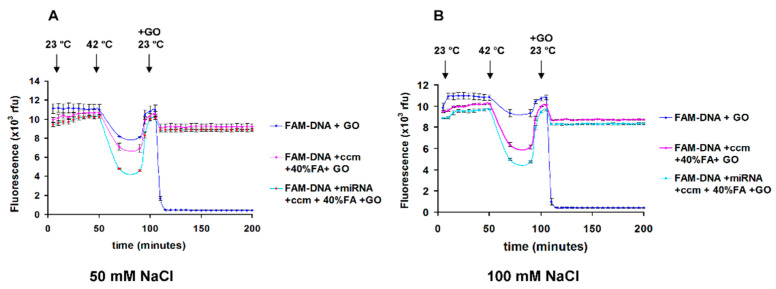
MicroRNA (18 nt) detection by fluorescence PET quenching in cell culture medium with increased ionic strength, (**A**) 50 mM NaCl, (**B**) 100 mM NaCl. The experiments were done in triplicate samples. Error bars represent the standard deviations of triplicate values.

Figure 5, highlight the microRNA (18 nt) target detection in the presence of 25% cell culture medium, 40% formamide concentration, 50 mM NaCl and 100 mM NaCl. The ionic strength of the diluted cell culture medium is 40 mM. The GO, 40 ug/mL, was added after hybridization (40 min incubation time at 42 °C) and then the signal recorded. The FAM-ssDNA: microRNA (18 nt) ratio is 1:1. The data was acquired first at 23 °C, at 42 °C and then at 23 °C. The cell culture medium, without Phenol Red, was pre-treated with 1% SDS and 0.1% Triton X-100. The hybridization is monitored using quenching by PET. FAM-DNA binding to GO is detected by fluorescence quenching due to FRET. The FAM-DNA + GO control sample contains 1% SDS, 0.1% Triton X-100, 0.1 mg/mL BSA, no formamide and no cell culture medium. 

The excess or unhybridized FAM-DNA fluorescent probe is bound to GO in the presence of 25% cell culture medium (ccm), 40% formamide, 1% SDS, 0.1% Triton X-100, 40 ug/mL GO, 8 nM microRNA (18nt) and 8 nM FAM-DNA. All molar ratios are 1:1, GO added after FAM-DNA/microRNA (18nt) hybridization. The experiments were done in triplicate samples.

The FAM-DNA binding to GO in complex medium containing salts (no cell culture medium and no formamide) is very fast and almost complete in about 5 min (FAM-DNA+GO, blue line). As shown in Figure 5, in the presence of cell culture medium, addition of GO resulted in ~15–20% fluorescence drop. After that, the signal became stable and no FAM-DNA adsorption was detected.

In the presence of cell culture medium and formamide the quenching by GO is decreased, but there is a difference in quenching between FAM-DNA + ccm + 40% FA + GO control sample and FAM-DNA + microRNA +ccm + 40% FA + GO probe sample. In this set up assay, any free unhybridized single stranded FAM-DNA will bind to GO. By using the 42 °C step and then 23 °C, we obtain also a less variable signal from the samples.

## 4. Discussion

Exosomes are membranous extracellular vesicles with diameters ranging from 30 to 150 nm that contain lipids, proteins, and nucleic acids [42]. On the other hand, unprotected DNA strands are rapidly degraded in serum due to nuclease activity and by adding SDS the DNA lifetime in serum is increased without disrupting DNA hybridization [43]. The use of surfactants allows exosome solubilization and increased detection sensitivity. By using SDS and formamide [39], nucleases containing cell culture medium are inactivated and microRNA is protected. In complex medium or cell culture medium, graphene oxide is maintained in dispersed form by SDS and Triton X-100 surfactants [44]. Prior to be used in assay mix, the GO was noncovalent modified with the anionic surfactant SDS and BSA. In cell culture medium GO quenching is decreased, but this property can be increased by adding 50 mM or 100 mM NaCl concentrations and we notice that the cell culture medium supplemented with platelet lysate shows autofluorescence that is quenched by GO (Figure S3). The optical detection is based on quenching by Förster resonance energy transfer from fluorescent DNA probe (FAM-ssDNA) to graphene oxide and PET quenching during specific hybridization, between fluorescent DNA probe and guanine base from complementary microRNA target. The quenchable dyes react as electron acceptors in the excited state and the guanine base interact as an electron donor. It was reported that 5-FAM may be quench by adenine base as well [16]. The quenching efficiencies (%) of nucleotides bases after hybridization, pairing with FAM fluorophore labeled oligo DNA are: adenine 23%, cytosine 8%, guanine 32%, thymine 8% [15]. Our 18 nucleotides out of 22 of microRNA-21 target, is used as a model biomarker. In order to measure the trace levels of microRNA, we optimized the hybridization conditions such as hybridization time, temperature, and graphene oxide dispersion in cell culture medium by the use of surfactants. The highest fluorescence quenching of the hybridized FAM-DNA/microRNA-21 was found when hybridization was conducted in a solution containing: 50% formamide, 0.1% SDS, 0.1% Triton X-100, 1 mM EDTA, 40 mM ionic strength, 10 mM Tris-HCl, pH 8.0 buffer at 23/42 °C for 30 min. The “pre-mixing” strategies, combining GO together with the probe FAM-DNA and a subsequent measurement of desorbed FAM-DNA hybridized to cDNA target, is a slow surface hybridization process [45,46] which add also difficulty in discriminating the signal contribution from the two significant quenching interactions PET and/or FRET towards the final measurement. 

## 5. Conclusions

We report label-free homogeneous FRET and PET detection of microRNA (18 nt) using graphene oxide and fluorescent DNA probes spiked in cell culture medium supplemented with platelet lysate. The “post-mixing” strategy is a more rapid solution-phase DNA hybridization measurement involving two steps, the first being DNA/microRNA hybridization detection by PET establishing its completion before addition of GO on the second step to preferentially adsorb unhybridized FAM-DNA probe. Subsequent GO-specific FRET quenching of unbound probe could then establish more accurate measurement of the probe-target complexes in solution. The use of sodium dodecyl sulfate to pre-treat cell culture medium was explored as a means to suppress nuclease activity without disrupting DNA/RNA hybridization; also, the formamide can suppress RNase activity. This amplification-free method uses a single step and mixed surfactant micelles treated cell culture medium. The “turn off” assay of microRNA (18 nt) is linear in the range of 1 nM and 8 nM in a single 100 uL total volume assay sample containing 25% cell culture medium supplemented with platelet lysate. This detection limits show the feasibility of both high-throughput and point-of-care clinical diagnostics. The results showed the potential of this homogeneous assay for fast, specific, and sensitive high-throughput analysis of low-abundance microRNAs in cell extracts, biofluids, and tissues.

## Supplementary Materials

The following are available online at https://www.mdpi.com/2079-4991/11/2/368/s1, Figure S1: microRNA (18 nt)/FAM-DNA hybrids quantitation by the intercalating dye PicoGreen, 30–50% formamide, 0.1% SDS, 0.1 mg/mL BSA, 23 °C; Figure S2: microRNA (18 nt)/FAM-DNA hybrids quantitation by the intercalating dye PicoGreen in the presence of, 30% formamide, 0.1% SDS, 0.1 mg/mL BSA, 23 °C; Figure S3: miRNA detection in cell culture medium in the presence of (**A**) 50 mM NaCl and (**B**) 100 mM NaCl.
