# Supplementary material for: Label-Free Homogeneous microRNA Detection in Cell Culture Medium Based on Graphene Oxide and Specific Fluorescence Quenching
Source: Nanomaterials (Basel). 2021 Feb 2;11(2):368. doi: 10.3390/nano11020368 (PMC7912907; doi:10.3390/nano11020368)
Supplement: Supplementary file 1 [file nanomaterials-11-00368-s001.pdf]

nanomaterials

Supplementary Materials

Label-Free Homogeneous microRNA Detection in Cell Culture Medium Based on Graphene Oxide and Specific Fluorescence Quenching

Florentin R. Nitu [1], Lorand Savu [2], Sorin Muraru [1], Ioan Stoian [3] and Mariana Ionită [1,*]

[1] Faculty of Medical Engineering, University Politehnica of Bucharest, Gh. Polizu St., no. 1-7, 011061 Bucharest, Romania; nitux001@umn.edu or fn@ddt.umn.edu or florentin.nitu@upb.ro (F.R.N.); sor.muraru@gmail.com (S.M.)

[2] Molecular Biology Department, Genetic Lab, Milcov Street, no. 5, Sector 1, 012244 Bucharest, Romania; office@geneticlab.ro

[3] Royal Hospital, Splaiul Unirii Street, no.313A, Sector 3, 030138 Bucharest, Romania; drstoianioan@royalhospital.ro

* Correspondence: mariana.ionita@polimi.it; Tel.: +40-21-4022709

One of the most used fluorescent dyes introduced into biomedical research are PicoGreen (PG) and SYBR Green I (SG) [1–4] due to the increase in their fluorescent emission upon interaction with double-stranded DNA (dsDNA). PicoGreen dsDNA quantitation is an ultra-sensitive fluorescent nucleic acid stain method for quantitating double-stranded DNA (dsDNA) in molecular biological procedures such as cDNA synthesis for library production and DNA fragment purification for subcloning, as well as diagnostic applications, such as quantitating DNA amplification products [1,5] and primer extension assays [6]. On binding DNA, PG fluorescence increases >1000-fold [3,7] and this is proportional to the quantity of DNA present. An interesting feature of PG is its ability to strongly bind not only to highly polymeric DNA but also to short duplexes <20 bp (base pairs), likewise exhibiting a sensitivity in the picogram range [7,8].

Alternate detection of oligo DNA-microRNA hybrid formation by fluorescent intercalating dye PicoGreen.

Figure 3, highlight the quantitation of microRNA (18 nt)/FAM-DNA hybrid by the intercalating dye PicoGreen, in the presence of bovine serum albumin, SDS, NaCl and 30%, 40% and 50% formamide concentration. We used an hybridization assay mixture containing: 0.1 mg/mL BSA, 0.1% SDS, 40 mM NaCl, 1 mM EDTA, 10 mM Tris-HCl pH 8.0, and four FAM-DNA concentrations: 1 nM, 2 nM, 4 nM, 8 nM, total assay volume is 100 uL. The microRNA (18 nt): FAM-ssDNA ratio is 1:1. The data was acquired at 23 °C. The hybridization is monitored using quenching by photoinduced electron transfer. This is an alternate method to prove microRNA/FAM-DNA hybridization in the presence of bovine serum albumin, SDS and salts. The data shows that the optimum binding of intercalating dye PicoGreen to microRNA/FAM-DNA hybrids is at 30% Formamide, 0.1 % SDS and 0.1 mg/mL BSA (orange line). At lower formamide concentration hybridization is decreased, while at higher concentration interfere with the binding of PicoGreen to double stranded hybrid RNA-DNA.

In Figure 1, the microRNA (18 nt) detection is plotted at the optimum binding of intercalating dye PicoGreen to microRNA/FAM-DNA hybrids: 30% Formamide, 0.1 % SDS and 0.1 mg/mL BSA (orange line in Figure 2).

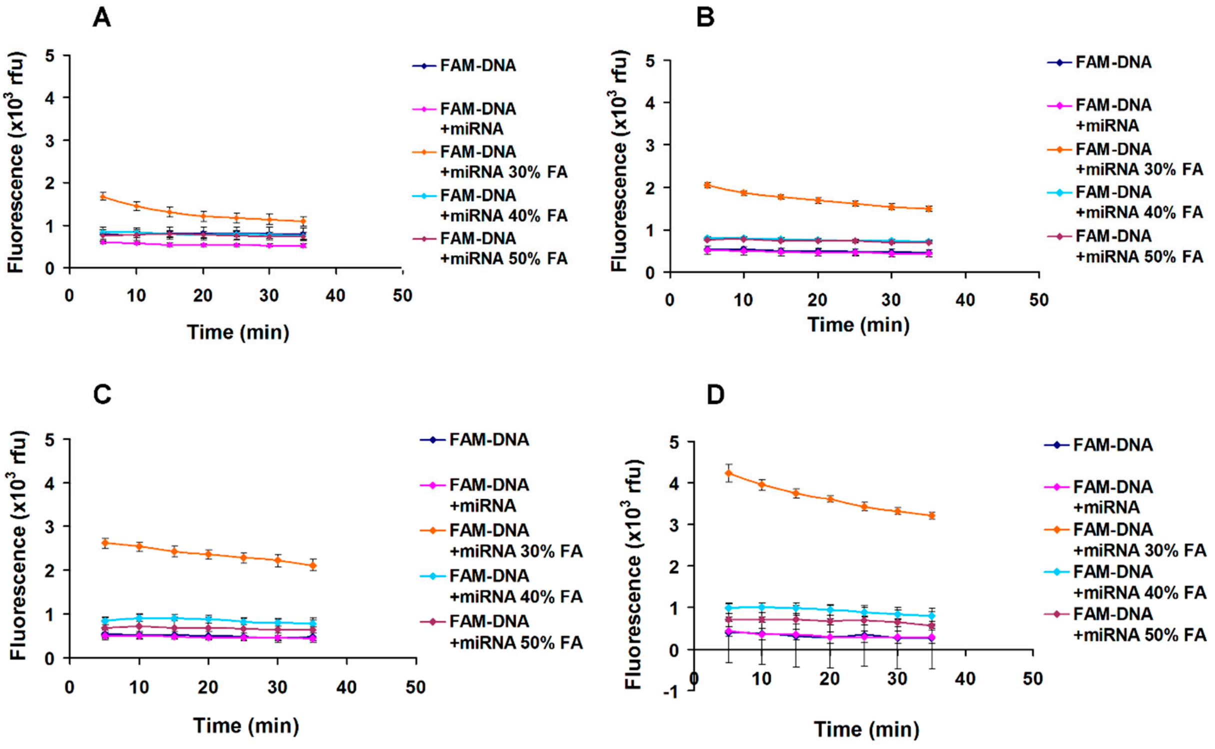

Figure S1. microRNA (18 nt)/FAM-DNA hybrids quantitation by the intercalating dye PicoGreen, 30–50% formamide, 0.1% SDS, 0.1 mg/mL BSA, 23 °C. (A) 1 nM microRNA (18 nt):FAM-DNA, (B) 2 nM microRNA (18 nt):FAM-DNA, (C) 4 nM microRNA (18 nt):FAM-DNA, (D) 8 nM microRNA (18 nt):FAM-DNA. All molar ratios are 1:1, PicoGreen added after FAM-DNA/microRNA 18 nt hybridization. The experiments were done in triplicate samples.

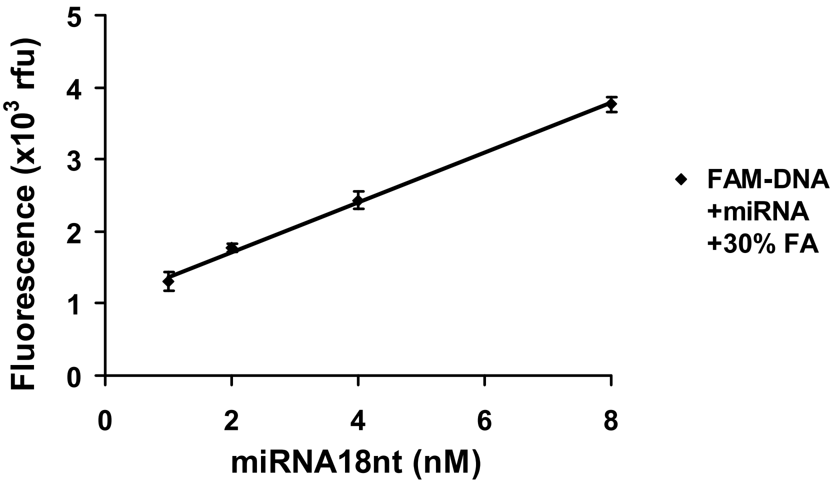

Figure S2. microRNA (18 nt)/FAM-DNA hybrids quantitation by the intercalating dye PicoGreen in the presence of, 30% formamide, 0.1% SDS, 0.1 mg/mL BSA, 23 °C. All molar ratios are 1:1, PicoGreen added after FAM-DNA/microRNA 18 nt hybridization. The experiments were done in triplicate samples.

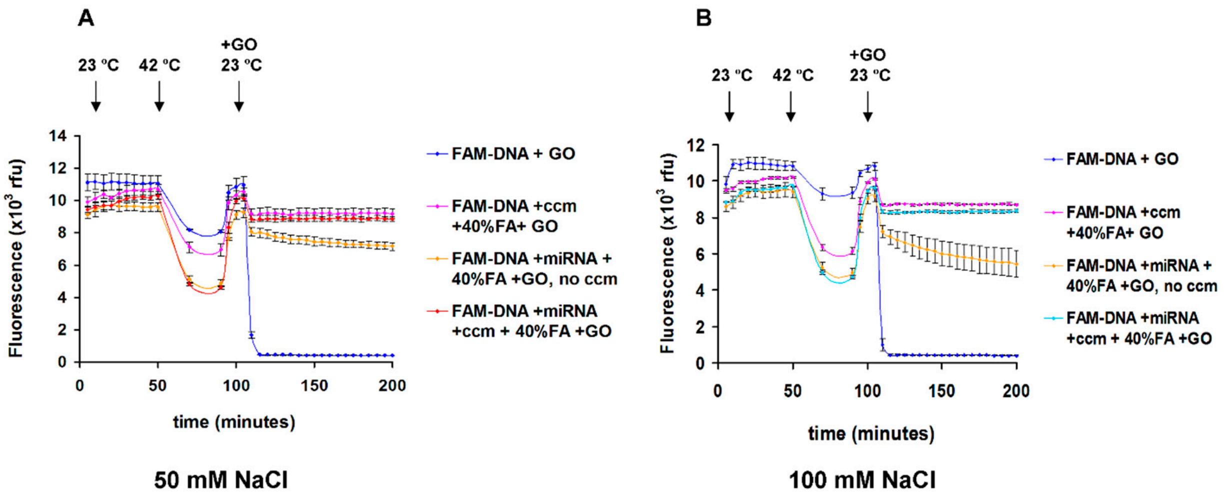

Figure S3. miRNA detection in cell culture medium in the presence of (**A**) 50 mM NaCl and (**B**) 100 mM NaCl. The experiments were done in triplicate samples.

References

1. Ahn; J, S.; Costa, J.; Emanuel, J.R. PicoGreen Quantitation of DNA: Effective Evaluation of Samples Pre-or Post-PCR. *Nucleic Acids Research*. **1996**, *24(13)*, p. 2623–2625.
2. Schneeberger, C.; Speiser, P.; Kury, F.; Zeillinger, R. Quantitative detection of reverse transcriptase-PCR products by means of a novel and sensitive DNA stain. *Genome Research*. **1995**, *4(4)*, p. 234–238.
3. Singer, V.L.; Jones, L.J.; Yue, S.T.; Haugland, R.P. Characterization of PicoGreen Reagent and Development of a Fluorescence-Based Solution Assay for Double-Stranded DNA Quantitation. *Analytical Biochemistry*, **1997**, *249(2)*, p. 228–238.
4. Zipper, H.; Brunner, H.; Bernhagen, J.; Vitzthum, F. Investigations on DNA intercalation and surface binding by SYBR Green I, its structure determination and methodological implications. *Nucleic Acids Research*. **2004**, *32(12)*, p. e103.
5. Enger, O. Use of the fluorescent dye PicoGreen for quantification of PCR products after agarose gel electrophoresis. *BioTechniques J2-Biotechniques*. **1996**, *21(3)*, p. 372–374.
6. Seville, M.; West, A.B.; Cull, M.G.; McHenry, C.S. Fluorometric Assay for DNA Polymerases and Reverse Transcriptase. *BioTechniques*. **1996**, *21(4)*, p. 664–672.
7. Dragan, A.I.; Casas-Finet, J.R.; Bishop, E.S.; Strouse, R.J.; Schenerman, M.A.; Geddes, C.D. Characterization of PicoGreen interaction with dsDNA and the origin of its fluorescence enhancement upon binding. *Biophysical journal*. **2010**, *99(9)*, p. 3010–3019.
8. Ikeda, Y; Iwakiri, S.; Yoshimori, T. Development and characterization of a novel host cell DNA assay using ultra-sensitive fluorescent nucleic acid stain "PicoGreen". *Journal of Pharmaceutical and Biomedical Analysis*. **2009**, *49(4)*, p. 997–1002.

© 2021 by the authors. Submitted for possible open access publication under the terms and conditions of the Creative Commons Attribution (CC BY) license (http://creativecommons.org/licenses/by/4.0/).
